# Safety and Efficiency of Cephalic Vein Puncture by Modified Seldinger Technique Compared to Subclavian Vein Puncture for Cardiac Implantable Electronic Devices

**DOI:** 10.1002/clc.24327

**Published:** 2024-07-30

**Authors:** Marie‐Christine Weidauer, Enzo Knüpfer, Jörg Lottermoser, Usama Alkomi, Steffen Schoen, Carsten Wunderlich, Marian Christoph, Alexander Francke

**Affiliations:** ^1^ Department of Cardiology Helios Klinikum Pirna Pirna Germany; ^2^ Department of Cardiology, TU Dresden Campus Chemnitz—MEDiC Klinikum Chemnitz Chemnitz Germany

**Keywords:** cardiac implantable electronic devices, cephalic vein puncture, subclavian vein puncture

## Abstract

**Introduction:**

The establishment of venous access is one of the driving factors for complications during implantation of pacemakers and defibrillators (cardiac implantable electronic devices [CIED]). Recently, a novel approach of accessing the cephalic vein for CIED by cephalic vein puncture (CVP) using a modified Seldinger technique has been described, promising high success rates and simplified handling with steeper learning curves. In this single‐center registry, we analyzed the safety and efficiency of CVP to SVP access after defining CVP as the primary access route in our center.

**Methods:**

A total of 229 consecutive patients receiving a CIED were included in the registry. Sixty‐one patients were implanted by primary or bail‐out SVP; 168 patients received primary cephalic preparation and CVP was performed when possible, using a hydrophilic transradial sheath.

**Results:**

Implantation of at least one lead via CVP was successful in 151 of 168 patients (90%), and implantation of all leads was possible in 122 of 168 patients (72.6%). Total implantation times and fluoroscopy times and doses did not differ between CVP and SVP implantations. Pneumothorax occurred in 0/122 patients implanted via CVP alone, but 8/107 (7.5%) patients received at least one lead via SVP.

**Conclusion:**

Our data confirms high success rates of the CVP for CIED implantation. Moreover, this method can be used without significantly prolonging the total procedure time or applying fluoroscopy dose compared to the highly efficient SVP while showing lower overall complication rates.

## Graphical abstract

Cephalic vein puncture has been described as novel access for CIED implantation, promising high success rates and simplified handling. Implantation of at least one lead via CVP was successful in 90% of the patients. Total implantation and fluoroscopy times did not differ between cephalic and subclavian implantations. Pneumothorax only occurred after subclavian puncture.

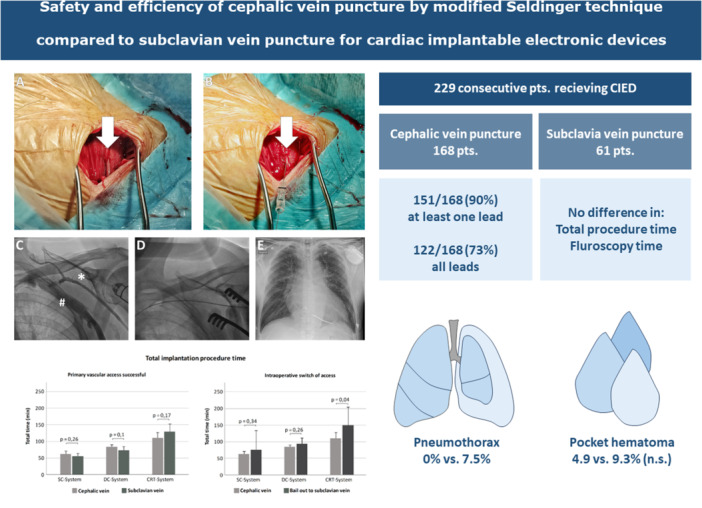

## 1 Introduction

Over the last decade, an increase in both the total number of implantations of cardiac implantable electronic devices (CIED) and the number of implanting centers in most member countries of the European Society of Cardiology has been recorded [1]. Considering this steadily growing number of procedures and implanting centers as well as the resulting large numbers of newly qualifying operators involved, the technique of venous access is crucial for optimum patient care. Therefore, the ideal venous access technique should be safe, quick to perform, and easy to learn with high success rates.

The current European Heart Rhythm Association (EHRA) expert consensus recommends cephalic vein access, or alternatively the extra‐thoracic puncture of the axillary vein (AVP), for primary access in all implantations [2]. The traditional proximal/central subclavian vein puncture (SVP) should only be performed in case of distal subclavian vein stenosis or occlusion when no other venous access is possible due to its unfavorable risk profile.

In terms of safety, the cephalic vein cut‐down (CVC) demonstrated superiority compared to SVP by avoiding potentially severe complications, such as pneumothorax and hemothorax, as well as reducing the risk for lead failure due to subclavian crush [2, 3, 4, 5]. However, CVC bears more technical and anatomical challenges and demands more experience and training of the surgeon.

Therefore, CVC access is not successful in all cases with a need to switch to an alternative, usually subclavian venous access [6, 7, 8]. Depending on the surgeons experience, this was required in 10%–40% of the patients with primarily cephalic approach [5, 7, 9]. This problem was previously addressed by proposing modifications to the original CVC such as the use of hydrophilic guidewires to account for steep cephalic‐subclavian entry angles or multiple guidewire techniques in conjunction with diagnostic catheters for wire manipulation [10, 11, 12]. Nevertheless, higher lead implantation failure rates of CVC are still the main reason for using SVP risking higher numbers of adverse events [2]. Concerns about the prolongation of procedure duration and slower learning curves for novel surgeons also trigger a preference for the seemingly more feasible SVP approach.

In contradiction to the current guideline recommendations, 59% of all German CIED implantations were performed via SVP according to mandatory German quality‐control data reported for all CIED implantations performed in 2019, as well as in 2020 [13].

Recently, a novel approach of accessing the cephalic vein for CIED by cephalic vein puncture (CVP) using a modified Seldinger technique instead of surgical CVC has been described, promising high success rates and simplified handling with steeper learning curves [14].

However, there currently is no direct comparison of CVP to SVP performance. In this single‐center prospective registry, we analyzed the safety and efficiency of CVP to SVP access after defining CVP as the primary access route.

## 2 Methods

### 2.1 Study Design

In this prospective single‐center registry analysis, the data of 229 consecutive patients who had undergone implantation of single‐ or dual‐chamber pacemaker (SC/DC PM), implantable cardioverter defibrillator (SC/DC ICD), or cardiac resynchronization therapy devices (CRT) were analyzed. All consecutive patients from 06/2020 to 09/2021 were included. There were no additional inclusion or exclusion criteria.

This study was approved by the ethics committee of Sächsische Landesärztekammer, Germany (EK‐BR‐115/20‐1) and was performed in accordance with the guidelines of good clinical practice. All patients had given informed consent to the procedure and partition in this registry.

All procedures were performed under continued antiplatelet therapy. Direct oral anticoagulants were paused on the day of the operation; in the case of coumarin treatment, an international normalized ratio (INR) of 2–2.5 was intended. Perioperative bridging was not performed.

### 2.2 CVP by Modified Seldinger Technique

Following intravenous administration of 1.5 g of Cefuroxime, the device implantation was performed under local anesthesia and conscious sedation with Midazolam and Fentanyl when necessary.

**Figure 1:**
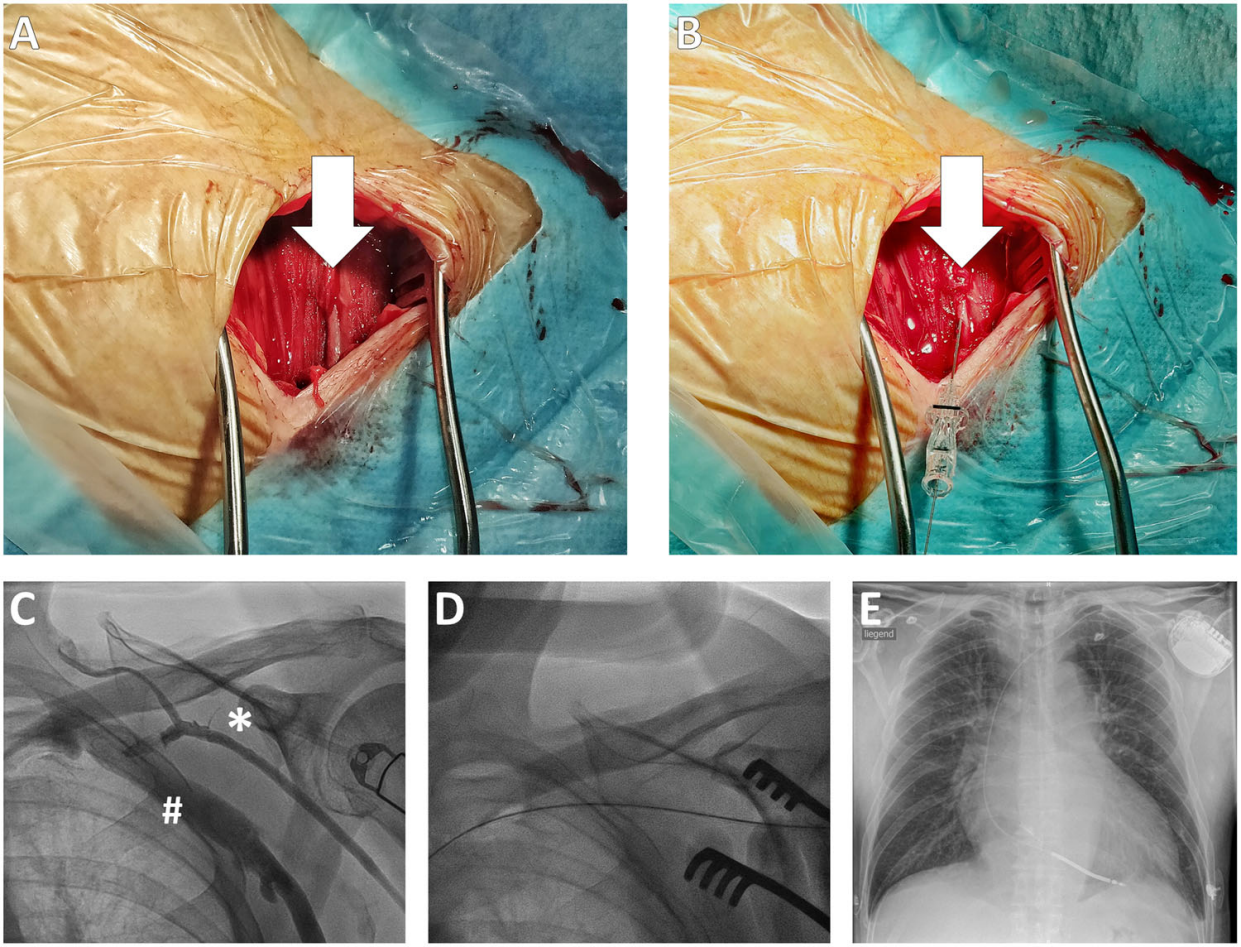
(A) Intraoperative preparation of the cephalic vein. (B) Direct puncture and guide‐wire insertion into the cephalic vein using the transradial introducer sheath. (C) Preoperative phlebography showing both the cephalic vein (*) and subclavian/axillary vein (#). (D) Guide‐wire inserted through the cephalic vein. (E) Postoperative chest X‐ray after successful SC‐ICD implantation through CVP.

In the study cohort, CVP was defined as the primary approach provided that a suitable vein was represented in the preprocedural contrast phlebography performed using an ipsilateral injection of 10 mL iodinated contrast agent. After skin incision and subcutaneous tissue dissection, approximately 1–2 cm of the course of the cephalic vein was exposed in the deltopectoral groove. As recently described, we then used a modified Seldinger technique to access the cephalic vein [14]. In contrast to the common venous cutdown technique, the vein was cannulated and splinted by using a transradial introducer sheath (Glidesheath Slender, 6 Fr, Transradial Kit, Terumo, Japan) to facilitate the insertion of up to three standard guidewires of the peel‐away sheaths [14]. Therefore, the cephalic vein was primarily punctured with a 22 Gauge needle, followed by inserting a 0.021 mini‐guidewire. Subsequently, the transradial introducer sheath was advanced over the mini‐guidewire. Up to three standard 0.035 guidewires were now introduced over the transradial introducer sheath. The process is shown in Figure 1.

In cases where the standard guidewire could not be advanced into the SVC, additional hydrophilic coronary guidewires or diagnostic catheters were used. Lead implantation was then performed using standard peel‐away sheaths. In the case of multiple lead implantation for DC or CRT systems, as many leads as possible were placed over the cephalic vein.

In cases of nonfeasibility of the cephalic venous access, the subclavian approach was used as a bail‐out strategy [2].

### 2.3 Aims

The aim of this study was to investigate the efficiency and safety of the CVP compared to SVP for CIED implantation after defining CVP as mandatory access for all procedures in a setting where most surgeons were not previously trained in cephalic vein access.

### 2.4 Primary Endpoints

The primary endpoints were the procedural success rate of CVP compared to SVP access, and the procedural duration as well as the fluoroscopy time and dose for CIED implantation.

### 2.5 Secondary Endpoints

The secondary endpoint was the incidence of short‐term complications, including pneumothorax, pericardial effusion, and bleeding complications in CVP compared to SVP. Additionally, learning curves with procedure times for newly trained surgeons were analyzed.

### 2.6 Statistical Analysis

Data was tested for normal distribution. Results of continuous variables are expressed as means ± standard deviation. Statistical analyses were performed using a two‐tailed, unpaired Student's *t* test for normally distributed data or the Mann–Whitney *U* test for nonparametric data. Analysis of variance was used when more than two classes were present. Adjustment for multiple testing was performed when necessary. Categorical variables are presented as total numbers with comparison using *χ* ^2^ statistics and Fisher exact test. The significance level was set to *p* < 0.05.

## 3 Results

### 3.1 Primary Endpoints—Efficiency of the Cephalic Vein Access by Modified Seldinger Technique

In total 229 consecutive patients who received CIED implantation between 06/2020 and 09/2021 were included in this study. The baseline characteristics of the cohort are presented in Table 1. Patient's demographic and clinical characteristics were comparable between the CVP and SVP groups. Overall, apreprocedural contrast phlebography was performed in 212 patients. According to the phlebography, in 168 out of 212 patients (79.3%) the cephalic vein presented with an anatomy suitable for CV access. As a bail‐out strategy, a primary subclavian vein access was used in 44 patients (20.8**%**).

**Table 1:** Baseline characteristics.

	Cephalic vein access (*n* = 168)	Subclavian vein access (*n* = 61)	*p* value
Age, years	74.7 (±9.8)	76.4 (±9.8)	0.24
Height, cm	170 (±10.3)	171 (±8.7)	0.46
Weight, kg	83.4 (±19.6)	78.5 (±16)	0.08
BMI	28.6 (±5.5)	26.6 (±5.1)	< 0.05
Male	112 (66.7)	40 (65.6)	0.88
Female	56 (33.3)	21 (34.4)	0.88
Heart failure (NYHA stage)			
No heart failure	29 (17.3)	4 (6.6)	< 0.05
NYHA 1	8 (4.8)	3 (4.9)	0.96
NYHA 2	82 (48.8)	37 (60.7)	0.11
NYHA 3	44 (26.2)	13 (21.3)	0.45
NYHA 4	5 (3)	4 (6.6)	0.22
Indications			
Pacemaker			
AV‐block	57 (33.9)	17 (27.9)	0.39
Sick sinus syndrome	41 (24.4)	17 (27.9)	0.59
Atrial fibrillation with bradyarrhythmia	9 (5.4)	7 (11.5)	0.11
Cardiomyopathy, LBBB, LVEF < 35%	19 (11.3)	8 (13.1)	0.71
ICD			
Primary prevention	29 (17.3)	3 (4.9)	< 0.05
Secondary prevention	12 (7.1)	8 (13.1)	0.16
Lead revision	1 (0.6)	1 (1.6)	0.45
Anticoagulation			
Antiplatelet (ASS, Clopidogrel, Prasugrel, Ticragrelor)	25 (14.9)	12 (19.7)	0.85
Antiplasmatic (Coumarin, direct‐oral anticoagulants, Heparin)	92 (54.7)	31 (50.8)	0.78
CIED system			
SC PM	13 (7.7)	10 (16.4)	< 0.05
DC PM	94 (56)	32 (52.5)	0.64
CRT‐PM	4 (2.4)	3 (4.9)	0.32
SC‐ICD	17 (10.1)	6 (9.8)	0.95
DC‐ICD	24 (14.3)	4 (6.6)	0.12
CRT‐ICD	16 (9.5)	6 (9.8)	0.94

Abbreviations: AV‐block, atrioventricular block; BMI, body mass index; CRT, cardiac resynchronization therapy; DC, dual chamber; ICD, implantable cardioverter defibrillator; LBBB, left bundle branch block; LVEF, left ventricular ejection fraction; PM, pacemaker; SC, single chamber.

In 17/212 (8%) of the preprocedurally performed contrast phlebographies, the cephalic vein could not be identified, was estimated too small for implantation (22/212, 10.4%) or presented with an unsuitable angle of confluence with the subclavian vein (4/212, 1.8%) (also see Supporting Information S1: Figure 1). An additional hydrophilic guidewire was used in 12.1% of the patients in the cephalic group versus 1.6% in the subclavian group. Cephalic vein access through puncture and placement of at least one lead was successful in 151 out of 168 patients (90%); an intraoperative bail‐out to the subclavian vein access for at least one lead was necessary in 46 out of 168 patients (27.4%) where cephalic vein access was primarily attempted (Table 2). The successful implantation of all leads via exclusive cephalic access could be performed in 72.6% of the cases when a cephalic approach was considered. With regard to the PM system, this applied to 27 of out 30 SC (90%) and 95 out of 118 (80.5%) DC devices. None of the CRT systems could be implanted through exclusive cephalic access.

**Table 2:** Procedural data.

Preprocedural venography of the cephalic vein (*n* = 212)	
Not identifiable	17/212 (8.0)
Small size	22/212 (10.3)
Branched anatomy	1/212 (0.5)
Unsuitable angle of confluence with axillary vein	4/212 (1.8)
Reasons for intraoperative switch to subclavian vein access (*n* = 168)	
Preparation of cephalic vein not possible	12/168 (7.1)
Introduction of 1st peel‐away sheath not possible	15/168 (8.9)
Introduction of 2nd peel‐away sheath not possible	6/168 (3.6)
Planned implantation of coronary‐sinus lead	13/168 (7.7)
Successful implantation of all leads using cephalic vein access	
Total	122/168 (72.6)
SC	27/30 (90.0)
DC	95/118 (80.5)
CRT	0/20 (0%)

Abbreviations: CRT, cardiac resynchronization therapy devices; DC, dual‐chamber devices; SC, single‐chamber devices.

**Figure 2:**
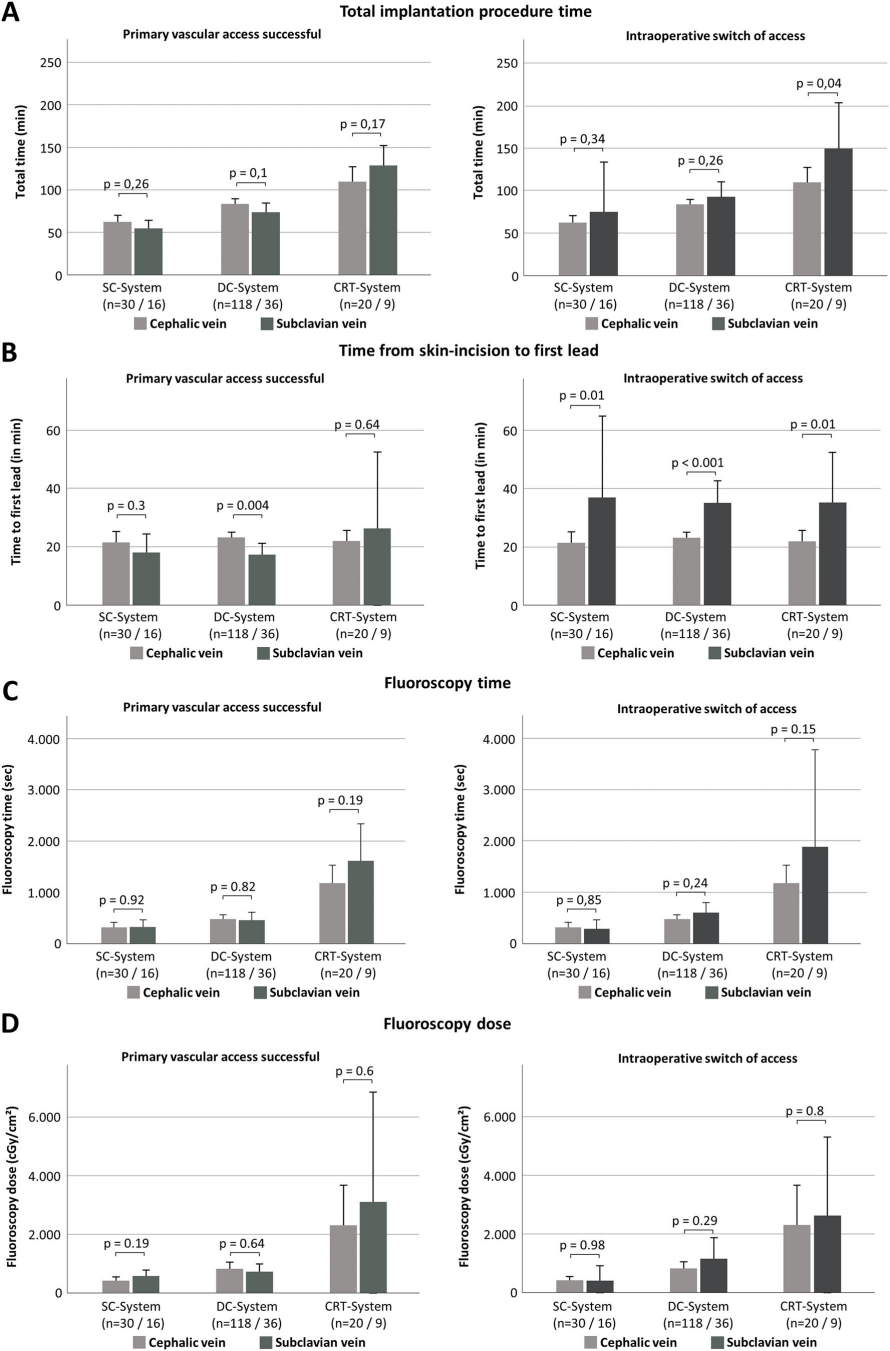
Procedural data comparing CIED implantation through CVP versus SVP depending on primarily intended SVP versus intraoperative switch to SVP after failed cephalic access. (A) Total implantion procedure time. (B) Skin‐to‐skin time. (C) Fluoroscopy time. (D) Fluoroscopy dose.

The total implantation time was not significantly different comparing the cephalic and subclavian access groups. With regard to the time between skin incision and successful implantation of the first lead, there was no significant difference between CVP and SVP among SC and CRT device implantation, while the cephalic vein access was more time‐consuming for DC device implantation in our cohort. When cephalic vein access was considered, but failed, bail‐out SVP access resulted in longer time to first lead implantation (Figure 2). Likewise, the total fluoroscopy time and dose did not differ significantly between the cephalic and the subclavian access groups.

### 3.2 Secondary Endpoints—Complications

The overall incidence of complications was lower in the cephalic access group compared to the subclavian group. Pneumothorax exclusively occurred in the subclavian access group (7.5%, *p* = 0.002). Furthermore, a numeric trend toward a higher number of pericardial effusions as well as bleeding complications was noticeable in the subclavian access group, though the difference was not statistically significant. There was no evidence of early device infection within the first 4 weeks after surgery in either of the access groups (Table 3).

**Table 3:** Complications.

	Cephalic vein access (*n* = 122)	Subclavian vein access (*n* = 107)	*p* value
Pneumothorax	0	8 (7.5)	0.002
Pericardial effusion	1 (0.8)	5 (4.7)	0.069
Pocket hematoma	6 (4.9)	10 (9.3)	0.19
Lead dislocation	0 (0)	0 (0)	0.25
Early device infection	0 (0)	0 (0)	0.25

Considering the learning curve of CVP puncture, two surgeons who previously exclusively performed SVP were newly trained in CVP and both have shown a significant improvement in access‐related procedure time over the course of their training as shown in Supporting Information S1: Figure 2.

## 4 Discussion

Here, we were able to show that changing the mandatory primary venous access for CIED from a subclavian puncture to the cephalic vein can be achieved without compromising procedure times or success rates.

CVP is as efficient as SVP considering procedure times and fluoroscopy, with high rates of success without additional risk of complications associated with SVP. Venous access by CVP is overall more feasible than the traditional CVC technique and allows steep learning curves for newly trained surgeons.

In our cohort, CVP and placement of at least one lead were successful in 90% of all patients with suitable anatomy documented by phlebography. Successful lead implantation of all leads was possible in 82% of the patients (122 out of 148) receiving SC and DC devices. Considering reported success rates for lead implantation using cephalic vein cutdown between 60% and 80% [7, 15, 16], our study shows that using CVP has the potential to improve these success rates.

The lower overall success rate in our cohort is mainly attributed to the inclusion of CRT device implantation. As a standard in our center, up to two leads are implanted via the cephalic vein primarily but due to its size, and the therefore potential risk of dislodging the other, already implanted leads, the left ventricular lead is a priori introduced using additional SVP. High success rates for implanting all three leads via cephalic vein access have previously been reported [5, 9, 17, 18]. However, with regard to short‐ and long‐term complications, this is still an issue of ongoing debate. Besides the risk of lead dislodgement in approximately 6% [18, 19], there might as well be a trend toward higher incidences of lead failure due to increased lead‐lead interactions [20, 21]. A future lead extraction tends to be more complicated if all leads are implanted via the same vein access [22].

Due to its higher success rates and technical simplicity, SVP was historically considered highly efficient for CIED implantation, especially for newly qualifying operators, which is why it was historically suggested as a primary access route [2].

Concerning procedural efficiency, the total implantation time is also of great interest, especially because longer procedure times are associated with an increased risk of infection [23]. Comparing the total procedural time of SVP and CVC, previous studies have shown inconsistent results. Longer procedural times for CVC are mostly attributed to the higher technical challenges of this technique [5, 7, 24]. However, due to its technical challenges, CVC is consequently highly dependent on the operator's experience. Reported shorter procedure times for CVC therefore most likely reflect experienced operators at high‐volume implanting centers [5, 9]. In centers with operators of different experience levels, it can be assumed that SVP is a less time‐consuming technique. In our cohort the total implantation time did not differ significantly between the cephalic and subclavian vein access group; however, there was a strong trend toward longer implantation times when cephalic access failed and SVP was used as a bail‐out—this was primarily triggered by longer time to first lead placement.

Furthermore, fluoroscopy times and doses are not significantly different between the different venous accesses even with regard to an intraoperative switch. These results imply that the CVP is noninferior to the highly efficient SVP considering total implantation time and fluoroscopy doses even if an intraoperative switch is necessary.

It should be added that we performed a preprocedural contrast venography before choosing the primarily attempted access. A cephalic vein suitable for attempting lead implantation was present in 79.3% of our patients, which is consistent with previous studies ranging from 69% to 88% [14, 25, 26, 27]. Anatomical challenges using cephalic vein access have previously been reported [25, 26]. Small vessel size, unsuitable anatomic course, or a cephalic vein not identifiable in the preprocedural contrast venography were the main reasons for primarily choosing the subclavian approach in our cohort. This data underlines the clinical importance of a preprocedural contrast venography.

In terms of complications, the superiority of the cephalic over subclavian vein access has been extensively proven [3, 6, 8]. Consistent with this data, pneumothorax only occurred in the subclavian access group in our cohort. Our data also suggests that CIED implantation can be performed under briefly paused anticoagulation without increased bleeding risk, regardless of the chosen vascular access.

In a recent development, the 2021 EHRA expert consensus paper recommends using axillary vein puncture (AVP) as a first‐line approach when cephalic vein access is not possible [2]. AVP combines high success rates of SVP with lower complication rates compared to SVP, especially with regard to the incidence of pneumothorax due to the extra‐thoracic puncture [7, 15, 16, 28, 29].

This can be even further reduced by using a 35° caudal fluoroscopic view while accessing the axillary vein [30]. The importance of a save extra‐thoracic puncture is underlined by the high rate of pneumothorax in our cohort when bail‐out subclavian puncture had to be performed.

When intraoperative phlebography is not possible due to renal failure, or the anatomy proves to be challenging, ultrasound‐guided AVP might further reduce complication rates and improve the overall outcome [31].

Considering the impact of all CIED‐associated complications on morbidity and mortality [32] and consequently also on health care costs [33], using the safest venous access for CIED implantation is crucial. Due to its safety profile cephalic vein access is considered the preferred approach for CIED implantation whereas AVP has not yet gained a widespread acceptance, despite its high level of recommendation [34].

In contrast to previous reports on CVC versus subclavian puncture, our data on CVP suggests high procedural success rates and steep learning curves for newly trained surgeons. Cephalic vein cutdown is arguably more challenging considering the surgical skill set of identifying, dissecting, and carefully cutting down the cephalic vein, and thereby might contribute toward the trend of using SVP as the seemingly faster and “safer” approach for newly trained surgeons. Training primarily on CVP reduced SVP access rates and thereby complication rates associated with this vascular access. We believe that CVP will reduce reservations toward cephalic vein access previously reported for CVC versus SVP [5, 7, 9].

### 4.1 Limitations

A direct comparison between the success rates for lead implantation between CVP and SVP is not possible because SVP is used as a bailout technique. CVP versus SVP was not compared in a randomized fashion, but in cases where the cephalic vein does not presented an suitable anatomy or lead implantation via CVP failed. Besides that, we did not compare CVP to AVP, which represents the currently equally recommended vein access according to its safety and efficiency profile. Therefore, a direct comparison between CVP and AVP should be performed in the future.

## 5 Conclusions

Our data shows the benefits of introducing CVP as standard access for CIED implantation. Moreover, this method can be used without significantly prolonging the total procedure time or applying fluoroscopy dose compared to the highly efficient SVP while showing lower overall complication rates. These results should encourage operators to primarily use a cephalic vein approach for CIED implantation.

## Ethics Statement

The study was approved by the ethics committee of Sächsische Landesärztekammer, Germany (EK‐BR‐115/20‐1) and was performed in accordance with the guidelines of good clinical practice.

## Consent

All patients had given informed consent to the procedure and partition in this registry.

## Conflicts of Interest

The authors declare no conflicts of interest.

## Supporting information

Supporting information.

## Data Availability

The data that support the findings of this study are available from the corresponding author upon reasonable request.

## References

[1] M. J. P. Raatikainen , D. O. Arnar , B. Merkely , et al., “A Decade of Information on the Use of Cardiac Implantable Electronic Devices and Interventional Electrophysiological Procedures in the European Society of Cardiology Countries: 2017 Report From the European Heart Rhythm Association,” Europace 19 (2017): ii1–ii90. 28903470 10.1093/europace/eux258

[2] H. Burri , C. Starck , A. Auricchio , et al., “Ehra Expert Consensus Statement and Practical Guide on Optimal Implantation Technique for Conventional Pacemakers and Implantable Cardioverter‐Defibrillators: Endorsed by the Heart Rhythm Society (HRS), the Asia Pacific Heart Rhythm Society (APHRS), and the Latin‐American Heart Rhythm Society (LAHRS),” Europace 23 (2021): 983–1008. 33878762 10.1093/europace/euaa367 PMC12378894

[3] A. P. Benz , M. Vamos , J. W. Erath , and S. H. Hohnloser , “Cephalic vs. Subclavian Lead Implantation in Cardiac Implantable Electronic Devices: A Systematic Review and Meta‐Analysis,” Europace 21 (2019): 121–129. 30020452 10.1093/europace/euy165

[4] F. Hasan , S. Nedios , Z. Karosiene , et al., “Perioperative Complications After Pacemaker Implantation: Higher Complication Rates With Subclavian Vein Puncture Than With Cephalic Vein Cutdown,” Journal of Interventional Cardiac Electrophysiology 66 (2022): 857–863. 35107720 10.1007/s10840-022-01135-x PMC10172219

[5] B. Ussen , P. S. Dhillon , L. Anderson , I. Beeton , M. Hickman , and M. M. Gallagher , “Safety and Feasibility of Cephalic Venous Access for Cardiac Resynchronization Device Implantation,” Pacing and Clinical Electrophysiology 34 (2011): 365–369. 21091741 10.1111/j.1540-8159.2010.02975.x

[6] V. Atti , M. K. Turagam , J. Garg , et al., “Subclavian and Axillary Vein Access Versus Cephalic Vein Cutdown for Cardiac Implantable Electronic Device Implantation,” JACC: Clinical Electrophysiology 6 (2020): 661–671. 32553216 10.1016/j.jacep.2020.01.006

[7] H. Calkins , B. M. Ramza , J. Brinker , et al., “Prospective Randomized Comparison of the Safety and Effectiveness of Placement of Endocardial Pacemaker and Defibrillator Leads Using the Extrathoracic Subclavian Vein Guided by Contrast Venography Versus the Cephalic Approach,” Pacing and Clinical Electrophysiology 24 (2001): 456–464. 11341082 10.1046/j.1460-9592.2001.00456.x

[8] I. Anagnostopoulos , C. Kossyvakis , M. Kousta , et al., “Different Venous Approaches for Implantation of Cardiac Electronic Devices. A Network Meta‐Analysis,” Pacing and Clinical Electrophysiology 45 (2022): 717–725. 35554947 10.1111/pace.14510

[9] I. Harding , N. Mannakkar , H. Gonna , et al., “Exclusively Cephalic Venous Access for Cardiac Resynchronisation: A Prospective Multi‐Centre Evaluation,” Pacing and Clinical Electrophysiology 43 (2020): 1515–1520. 32860243 10.1111/pace.14046

[10] T. M. Kolettis , D. N. Lysitsas , D. Apostolidis , G. G. Baltogiannis , E. Sourla , and L. K. Michalis , “Improved ‘Cut‐Down’ Technique for Transvenous Pacemaker Lead Implantation,” Europace 12 (2010): 1282–1285. 20519193 10.1093/europace/euq173

[11] J. P. Camous , F. Raybaud , I. Lesto , and P. H. Benoit , “Introduction of Permanent Cardiac Stimulation/Defibrillation Leads via the Retro‐Pectoral Veins,” Pacing and Clinical Electrophysiology 28 (2005): 324–325. 15826267 10.1111/j.1540-8159.2005.09400.x

[12] R. Neri , A. S. Cesario , D. Baragli , et al., “Permanent Pacing Lead Insertion Through the Cephalic Vein Using an Hydrophilic Guidewire,” Pacing and Clinical Electrophysiology 26 (2003): 2313–2314. 14675018 10.1111/j.1540-8159.2003.00365.x

[13] “Anual Report of the German Institute of Quality Control and Transparency in Health Care,” IQTIG, 2020, https://iqtig.org/qs-verfahren/hsm/.

[14] L. M. Rademakers and F. A. Bracke , “Cephalic Vein Access by Modified Seldinger Technique for Lead Implantations,” Pacing and Clinical Electrophysiology 44 (2021): 607–613. 33609409 10.1111/pace.14200

[15] J. Jiménez‐Díaz , F. Higuera‐Sobrino , J. Piqueras‐Flores , P. Pérez‐Díaz , and M. A. González‐Marín , “Fluoroscopy‐Guided Axillary Vein Access vs Cephalic Vein Access in Pacemaker and Defibrillator Implantation: Randomized Clinical Trial of Efficacy and Safety,” Journal of Cardiovascular Electrophysiology 30 (2019): 1588–1593. 31310038 10.1111/jce.14060

[16] N.‐Y. Chan , N.‐P. Kwong , and A.‐P. Cheong , “Venous Access and Long‐Term Pacemaker Lead Failure: Comparing Contrast‐Guided Axillary Vein Puncture With Subclavian Puncture and Cephalic Cutdown,” Europace 19 (2016): euw147. 10.1093/europace/euw147 27733455

[17] J. Vogler , A. Geisler , N. Gosau , et al., “Triple Lead Cephalic Versus Subclavian Vein Approach in Cardiac Resynchronization Therapy Device Implantation,” Scientific Reports 8 (2018): 17709. 30532064 10.1038/s41598-018-35994-0 PMC6286359

[18] A. Hadjis , R. Proietti , and V. Essebag , “Implantation of Cardiac Resynchronization Therapy Devices Using Three Leads by Cephalic Vein Dissection Approach,” Europace 19 (2017): 1514–1520. 28340223 10.1093/europace/euw276 PMC5834013

[19] J. B. van Rees , M. K. de Bie , J. Thijssen , C. J. W. Borleffs , M. J. Schalij , and L. van Erven , “Implantation‐Related Complications of Implantable Cardioverter‐Defibrillators and Cardiac Resynchronization Therapy Devices,” Journal of the American College of Cardiology 58 (2011): 995–1000. 21867832 10.1016/j.jacc.2011.06.007

[20] C. R. Barbhaiya , O. Niazi , J. Bostrom , et al., “Early ICD Lead Failure in Defibrillator Systems With Multiple Leads via Cephalic Access,” Journal of Cardiovascular Electrophysiology 31 (2020): 1462–1469. 32356380 10.1111/jce.14523

[21] Z. Akhtar , I. Harding , A. I. Elbatran , et al., “Multi‐Lead Cephalic Venous Access and Long‐Term Performance of High‐Voltage Leads,” Journal of Cardiovascular Electrophysiology 32 (2021): 1131–1139. 33565195 10.1111/jce.14939

[22] P. Syska , “Cephalic Access With Multiple Leads May Increase the Risk of Early ICD Lead Failure. Time to Question the Dogma?” Journal of Cardiovascular Electrophysiology 31 (2020): 1470–1471. 32356394 10.1111/jce.14522

[23] K. A. Polyzos , A. A. Konstantelias , and M. E. Falagas , “Risk Factors for Cardiac Implantable Electronic Device Infection: A Systematic Review and Meta‐Analysis,” Europace 17 (2015): 767–777. 25926473 10.1093/europace/euv053

[24] A. Nocito , S. Wildi , K. Rufibach , P.‐A. Clavien , and M. Weber , “Randomized Clinical Trial Comparing Venous Cutdown With the Seldinger Technique for Placement of Implantable Venous Access Ports,” British Journal of Surgery 96 (2009): 1129–1134. 19731229 10.1002/bjs.6730

[25] B. P. Knight , K. Curlett , H. Oral , F. Pelosi , F. Morady , and S. A. Strickberger , “Clinical Predictors of Successful Cephalic Vein Access for Implantation of Endocardial Leads,” Journal of Interventional Cardiac Electrophysiology 7 (2002): 177–180. 12397228 10.1023/a:1020893923079

[26] T. Tokano , Y. Nakazato , T. Shiozawa , et al., “Variations in Cephalic Vein Venography for Device Implantation–Relationship to Success Rate of Lead Implantation,” Journal of Arrhythmia 29 (2013): 9–12.

[27] H.‐F. Tse , C.‐P. Lau , and S.‐K. Leung , “A Cephalic Vein Cutdown and Venography Technique to Facilitate Pacemaker and Defibrillator Lead Implantation,” Pacing and Clinical Electrophysiology 24 (2001): 469–473. 11341084 10.1046/j.1460-9592.2001.00469.x

[28] F. Migliore , M. Siciliano , M. De Lazzari , et al., “Axillary Vein Puncture Using Fluoroscopic Landmarks: A Safe and Effective Approach for Implantable Cardioverter Defibrillator Leads,” Journal of Interventional Cardiac Electrophysiology 43 (2015): 263–267. 25956478 10.1007/s10840-015-0011-7

[29] F. Migliore , A. Curnis , and E. Bertaglia , “Axillary Vein Technique for Pacemaker and Implantable Defibrillator Leads Implantation: A Safe and Alternative Approach?” Journal of Cardiovascular Medicine 17 (2016): 309–313. 25252042 10.2459/JCM.0000000000000154

[30] F. Yang and G. Kulbak , “A New Trick to a Routine Procedure: Taking the Fear Out of the Axillary Vein Stick Using the 35° Caudal View,” Europace 17 (2015): 1157–1160. 25969438 10.1093/europace/euv066

[31] F. Migliore , L. Fais , R. Vio , et al., “Axillary Vein Access for Permanent Pacemaker and Implantable Cardioverter Defibrillator Implantation: Fluoroscopy Compared to Ultrasound,” Pacing and Clinical Electrophysiology 43 (2020): 566–572. 32394452 10.1111/pace.13940

[32] P. Palmisano , F. Guerra , G. Dell'Era , et al., “Impact on All‐Cause and Cardiovascular Mortality of Cardiac Implantable Electronic Device Complications,” JACC: Clinical Electrophysiology 6 (2020): 382–392. 32327071 10.1016/j.jacep.2019.11.005

[33] D. J. Cantillon , D. V. Exner , N. Badie , et al., “Complications and Health Care Costs Associated With Transvenous Cardiac Pacemakers in a Nationwide Assessment,” JACC: Clinical Electrophysiology 3 (2017): 1296–1305. 29759627 10.1016/j.jacep.2017.05.007

[34] M. G. Bongiorni , A. Proclemer , D. Dobreanu , et al., “Preferred Tools and Techniques for Implantation of Cardiac Electronic Devices in Europe: Results of the European Heart Rhythm Association Survey,” Europace 15 (2013): 1664–1668. 24170423 10.1093/europace/eut345

