# Supplementary material for: Safety and Efficiency of Cephalic Vein Puncture by Modified Seldinger Technique Compared to Subclavian Vein Puncture for Cardiac Implantable Electronic Devices
Source: Clin Cardiol. 2024 Jul 30;47(8):e24327. doi: 10.1002/clc.24327 (PMC11287195; doi:10.1002/clc.24327)
Supplement: Supplementary file 1 — Supporting information. [file CLC-47-e24327-s001.docx]

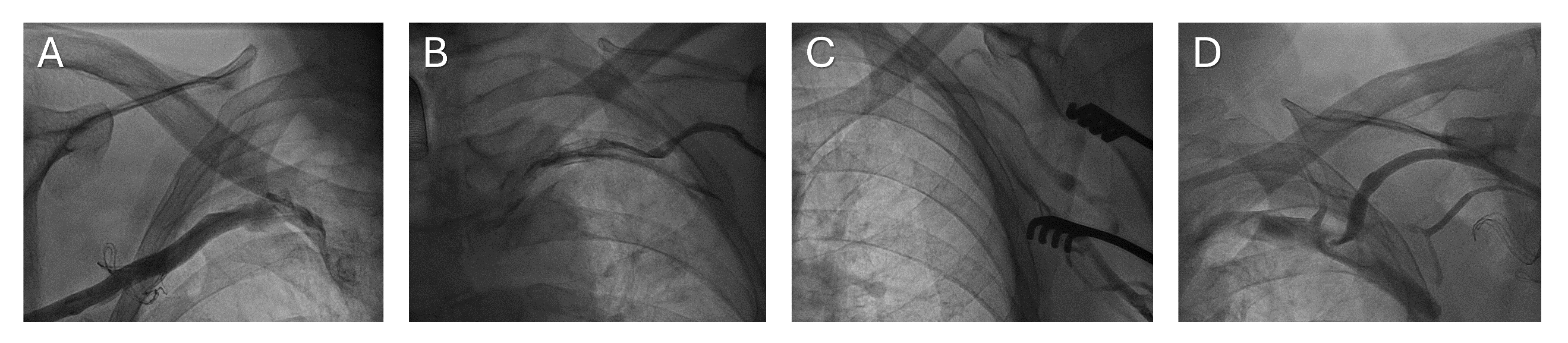


**Supplementary Figure 1:**

**Preoperative phlebography showing unsuitable anatomies for cephalic approach**

A: not identifiable, B: small size, C: branched anatomy (showing two small cephalic veins) D:

Unsuitable angle of confluence with axillary vein


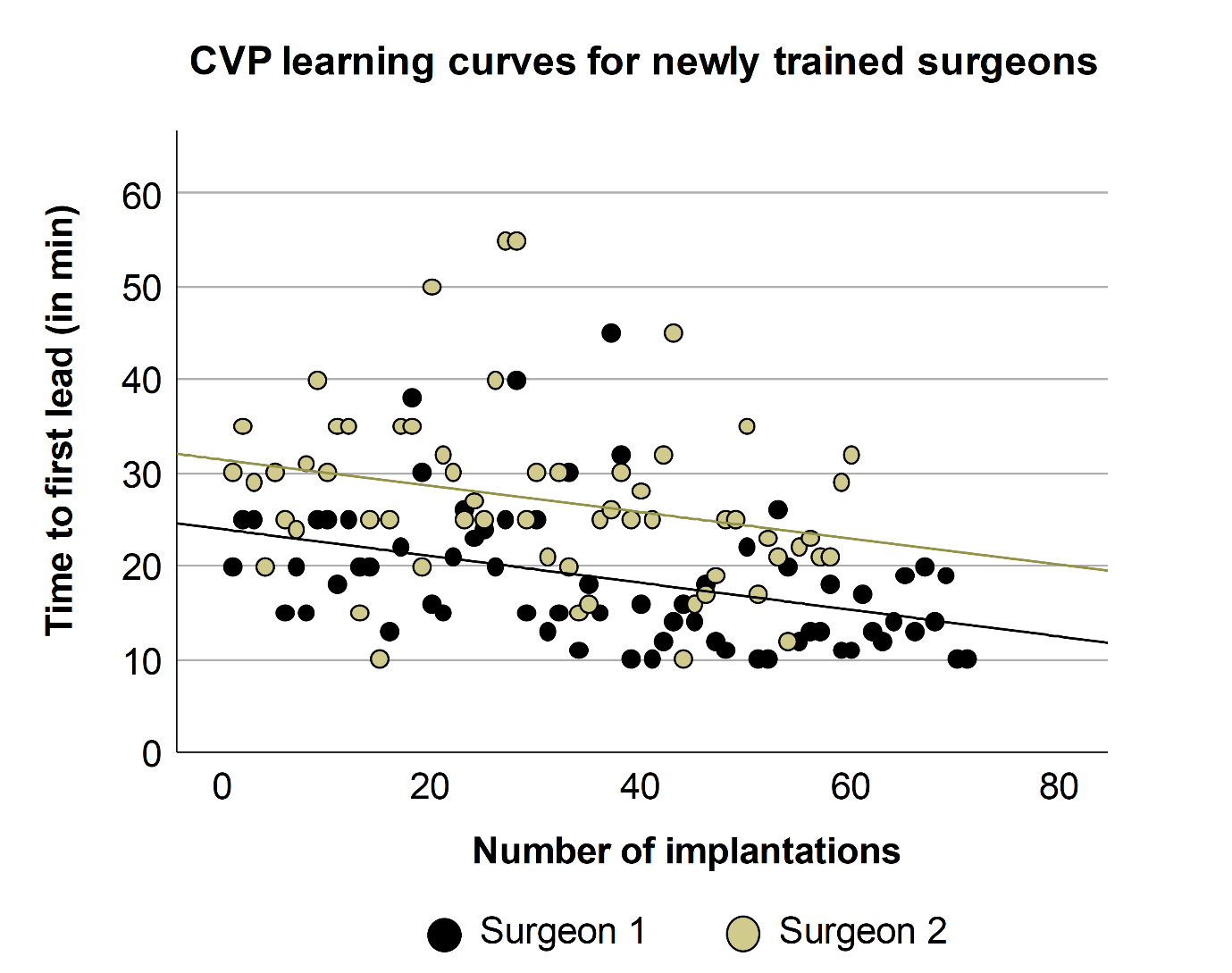


**Supplementary Figure 2**

Learning curves over time for 2 surgeons that had previously never performed CIED implantations through CVP, showing improvement in vascular access-related procedure time over the course of their training.
